# PNPLA3 variant and portal/periportal histological pattern in patients with biopsy-proven non-alcoholic fatty liver disease: a possible role for oxidative stress

**DOI:** 10.1038/s41598-017-15943-z

**Published:** 2017-11-17

**Authors:** Guido Carpino, Daniele Pastori, Francesco Baratta, Diletta Overi, Giancarlo Labbadia, Licia Polimeni, Alessia Di Costanzo, Gaetano Pannitteri, Roberto Carnevale, Maria Del Ben, Marcello Arca, Francesco Violi, Francesco Angelico, Eugenio Gaudio

**Affiliations:** 1 Department of Movement, Human and Health Sciences, University of Rome “Foro Italico”, Rome, Italy; 2 Department of Internal Medicine and Medical Specialties, I Clinica Medica, Sapienza University of Rome, Rome, Italy; 3 Department of Anatomical, Histological, Forensic Medicine and Orthopaedics Sciences, Sapienza University, Rome, Italy; 4 Department of Cardiovascular, Respiratory, Nephrologic, Anaesthesiologic and Geriatric Sciences, Sapienza University of Rome, Rome, Italy; 5 Department of Medical-Surgical Sciences and Biotechnologies, Sapienza University of Rome, Latina, Italy; 6 Department of Public Health and Infectious Diseases, Sapienza University, Rome, Italy

## Abstract

Pathogenesis of non-alcoholic fatty liver disease (NAFLD) is influenced by predisposing genetic variations, dysmetabolism, systemic oxidative stress, and local cellular and molecular cross-talks. Patatin-like phospholipase domain containing 3 (PNPLA3) gene I148M variant is a known determinant of NAFLD. Aims were to evaluate whether PNPLA3 I148M variant was associated with a specific histological pattern, hepatic stem/progenitor cell (HpSC) niche activation and serum oxidative stress markers. Liver biopsies were obtained from 54 NAFLD patients. The activation of HpSC compartment was evaluated by the extension of ductular reaction (DR); hepatic stellate cells, myofibroblasts (MFs), and macrophages were evaluated by immunohistochemistry. Systemic oxidative stress was assessed measuring serum levels of soluble NOX2-derived peptide (sNOX2-dp) and 8-isoprostaglandin F_2α_ (8-iso-PGF_2α_). PNPLA3 carriers showed higher steatosis, portal inflammation and HpSC niche activation compared to wild-type patients. DR was correlated with NAFLD activity score (NAS) and fibrosis score. Serum 8-iso-PGF_2α_ were significantly higher in I148M carriers compared to non-carriers and were correlated with DR and portal inflammation. sNox2-dp was correlated with NAS and with HpSC niche activation. In conclusion, NAFLD patients carrying PNPLA3 I148M are characterized by a prominent activation of HpSC niche which is associated with a more aggressive histological pattern (portal fibrogenesis) and increased oxidative stress.

## Introduction

Non-alcoholic fatty liver disease (NAFLD) represents the most common form of chronic liver disease worldwide^1^. NAFLD includes a spectrum of diseases ranging from simple fatty liver to non-alcoholic steatohepatitis (NASH) and may progress to cirrhosis and hepatocellular carcinoma^1^. The pathogenesis of NAFLD is multifactorial and could be influenced by predisposing genetic variations, dysmetabolic conditions, systemic oxidative stress, and local cellular and molecular cross-talks^2^.

The single nucleotide polymorphism in residue 148 (I148M, rs738409) in human patatin-like phospholipase domain containing 3 (PNPLA3) gene, which exhibits a C-to-G transition resulting in an amino acid substitution of isoleucine to methionine, is one of the strongest genetic determinants of NAFLD^3–5^. This mutation was suggested to impair triglyceride hydrolysis and potentially explain the increased triglyceride accumulation in hepatocytes^4^. Moreover, PNPLA3 variants have been associated with a worse histological depict in NAFLD^6^; however, no studies investigated the potential influence of PNPLA3 I148M variant in the development of specific histological pattern and in the intricate cross-talks between different cellular compartments activated by the regenerative response to liver damage in NAFLD.

In fact, the progression of NAFLD is determined by interactions between resident and recruited cells^7^. Triglyceride accumulation and the consequent long-lasting oxidative stress induce apoptosis and cell cycle arrest in hepatocytes, thus triggering the activation of the facultative Hepatic stem/progenitor cell (HpSC) niche^8,9^. The HpSC niche is composed by macrophages, Hepatic Stellate Cells (HSCs), and well-defined extracellular matrix compounds. Remarkably, HpSC response is influenced by macrophage subsets^10–13^; in turn, HpSC proliferation can activate HSCs, thus participating in fibrogenesis and leading to portal fibrosis^14^.

Therefore, the aims of the present study have been to evaluate whether NAFLD patients carrying PNPLA3 I148M variant: i) showed a specific histological pattern at liver biopsy; ii) were characterized by a prominent activation of HpSC niche; iii) presented increased levels of serum systemic oxidative stress markers.

## Results

### Anthropometric, clinical and genetic parameters

Anthropometric and clinical characteristics of patients are listed in Table 1. Mean age was 48.6 ± 12.4 years, and 48.1% were women. Mean Body Mass Index (BMI) was 29.3 ± 4.2 Kg/m^2^, 47.5% suffered from arterial hypertension and 40.0% from diabetes. As regard PNPLA3, 39/54 (72%) patients carried at least one allele with the I148M variant while 15/54 (28%) patients have a wild-type (WT) genotype. Patients with and without PNPLA3 I148M variant had similar characteristics, with the exception for arterial hypertension, which was less prevalent in patients with I148M variant (Table 1).

**Table 1:** Clinical characteristics of NAFLD patients according to the PNPLA3 genotype.

	NAFLD (n = 54)	Wild type (CC carrier) (n = 15)	PNPLA3 (GC/GG variant carrier) (n = 39)	*p-value*
Age (years)	48.6 ± 12.4	51.7 ± 47.3	47.3 ± 12.6	0.307
Women (%)	48.1	40.0	51.3	0.731
Current cigarette smokers (%)	32.5	33.3	32.1	1.000
Arterial hypertension (%)	47.5	83.3	32.1	**<0**.**05**
Diabetes Mellitus (%)	40.0	25.0	46.4	0.297
Body Mass Index (kg/m^2^)	29.3 ± 4.2	29.0 ± 3.5	29.4 ± 4.5	0.785
Waist circumference (cm)	104.1 ± 10.3	103.7 ± 9.9	104.2 ± 10.7	0.889
Metabolic syndrome (%)	59.0	60.0	59.0	0.598
Fasting plasma glucose (mg/dl)	105.9 ± 36.4	96.7 ± 16.5	110.1 ± 42.2	0.295
Total cholesterol (mg/dl)	195.3 ± 36.8	189.9 ± 38.7	197.6 ± 36.6	0.569
Low density lipoprotein (mg/dl)	116.1 ± 30.6	111.4 ± 40.9	117.9 ± 26.4	0.574
High density lipoprotein (mg/dl)	48.5 ± 22.8	43.0 ± 9.0	50.7 ± 26.3	0.350
Triglycerides (mg/dl)	157.4 ± 103.0	163.2 ± 72.8	155.1 ± 114.1	0.830
Statin (%)	19.4	16.7	20.8	1.000
Aspartate aminotransferase (U/l)	49.9 ± 33.1	39.2 ± 17.7	54.4 ± 37.2	0.089
Alanine aminotransferase (U/l)	82.2 ± 48.2	62.2 ± 32.9	90.8 ± 51.6	**<0**.**05**
GGT (U/l)	71.4 ± 57.0	74.2 ± 79.7	70.2 ± 46.0	0.845
Creatinine (mg/dl)	0.8 ± 0.2	0.8 ± 0.1	0.8 ± 0.2	0.699
sNox2-dp (pg/ml)	37.2 ± 15.0	33.8 ± 10.3	38.7 ± 16.6	0.270
Serum F2-Isoprostanes (pg/ml)	70.1 ± 11.7	64.4 ± 14.8	72.5 ± 9.4	**<0**.**05**

Data are reported as mean ± standard deviation. *p value* in bold are statistically significant. NAFLD: nonalcoholic fatty liver disease; PNPLA3: patatin-like phospholipase domain containing 3; GGT: Gamma-glutamyltranspeptidase; sNox2-dp: soluble Nox2-derived peptide.

### Histo-pathological analysis

**Figure 1:**
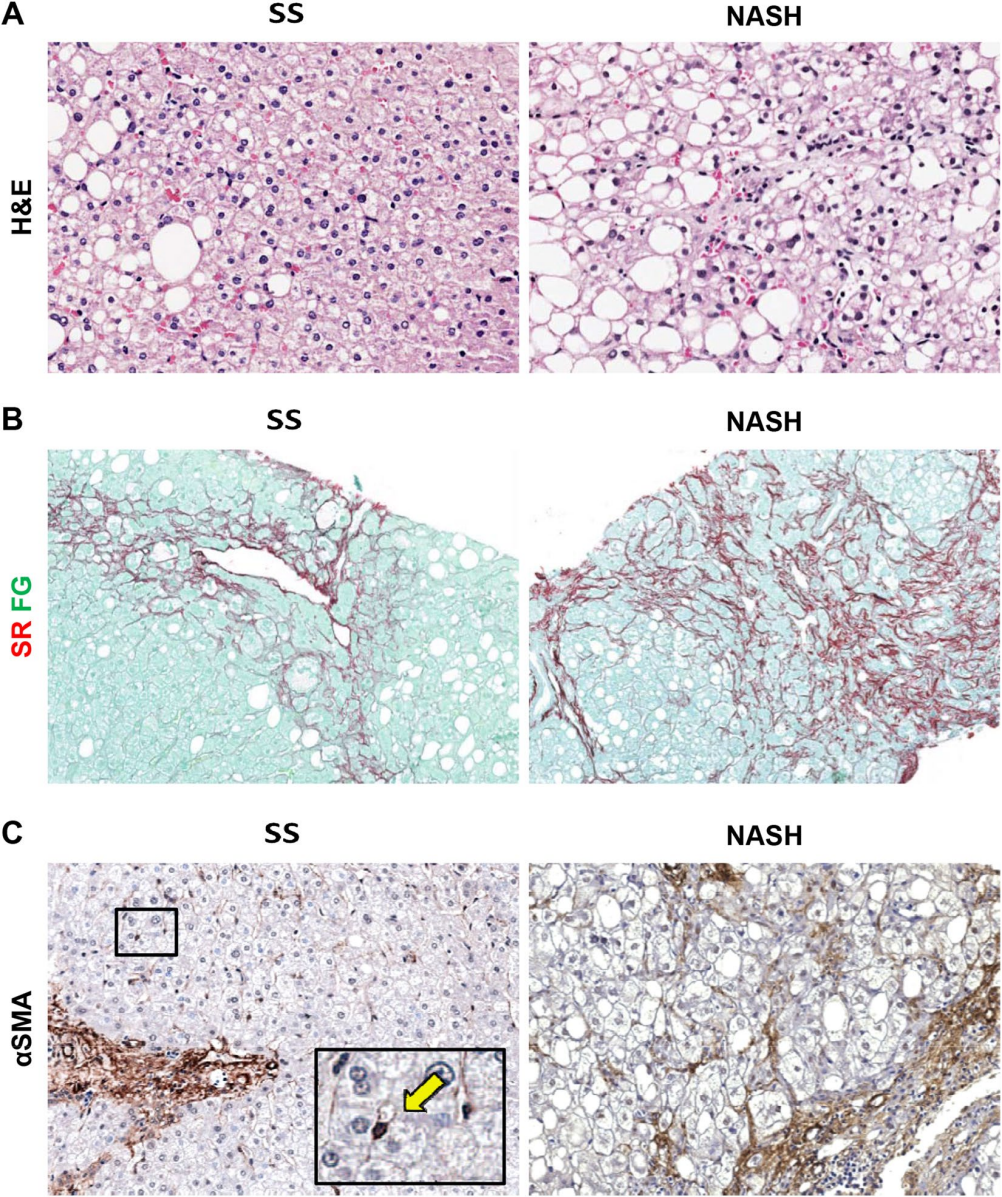
Histo-pathological evaluation and fibrosis in non-alcoholic fatty liver disease biopsies. (**A**,**B**) Hematoxylin & Eosin (H&E) stain in A and Sirius Red/Fast green (SR/FG) stain in B in simple steatosis (SS) and definite steatohepatitis (NASH). NASH biopsies were characterized by a higher inflammation and fibrosis in comparison with SS. Original Magnification (OM) = 20x (**A**) and 10x (**B**). (**C**) Immunohistochemistry for α smooth muscle actin (αSMA). Higher number of αSMA-positive stellate cells/myofibroblasts was revealed in NASH when compared with SS biopsies. OM = 20x. Single hepatic stellate cell was magnified in the box (arrow).

Liver biopsies were classified into SS (simple steatosis; N = 25), NASH (definite steatohepatitis; N = 25), and borderline, zone 3 pattern (N = 4). The NAFLD Activity Score (NAS) ranged from 1 to 8. Fibrosis of some degree was seen in 51/54 biopsy samples: stage 1 in 13 samples, stage 2 in 21, stage 3 in 14, and stage 4 in 3. Portal inflammation was seen in 26/54 biopsy samples: 8/25 (32%) SS biopsies and in 16/25 (64%) NASH biopsies (*p* = 0.023). Biopsies with NASH showed significantly higher steatosis, inflammation, hepatocellular ballooning, and NAS score when compared to biopsies with SS (Table 2, Fig. 1). Furthermore, NASH biopsies showed higher fibrosis score when compared to SS biopsies (Table 2 and Fig. 1). NAS score was directly correlated with fibrosis score (*r* = 0.484; *p* = 0.000).

**Table 2:** Histological and immunohistochemical findings.

	SS (n = 25)	NASH (n = 25)	*p-value*
NAFLD activity score	2.73 ± 1.00	5.19 ± 1.18	**0**.**000**
Steatosis	1.08 ± 0.89	2.07 ± 0.87	**0**.**000**
Lobular inflammation	0.73 ± 0.53	1.63 ± 0.63	**0**.**000**
Ballooning	0.92 ± 0.80	1.48 ± 0.75	**0**.**006**
Portal inflammation (%)	32.0	64.0	**0**.**023**
Fibrosis score	1.38 ± 0.75	2.59 ± 0.80	**0**.**000**
Ductular Reaction	0.15 ± 0.13	0.50 ± 0.59	**0**.**005**
EpCAM+ hepatocytes	0.39 ± 0.58	0.96 ± 0.77	**0**.**004**
Pericentral αSMA+ HSCs	3.81 ± 3.86	6.39 ± 4.58	**0**.**024**
Portal/septal αSMA+ MFs	4.41 ± 3.47	7.09 ± 5.74	**0**.**034**
Lobular S100A9+ macrophages	8.80 ± 5.46	7.11 ± 3.78	0.113
Portal S100A9+ macrophages	2.51 ± 1.70	5.43 ± 3.72	**0**.**002**

Data are reported as mean ± standard deviation. *p value* in bold are statistically significant. SS: simple steatosis; NASH: nonalcoholic steatohepatitis; HSCs: hepatic stellate cells; MFs: myofibroblasts; αSMA: α smooth muscle actin.

### HpSC activation

**Figure 2:**
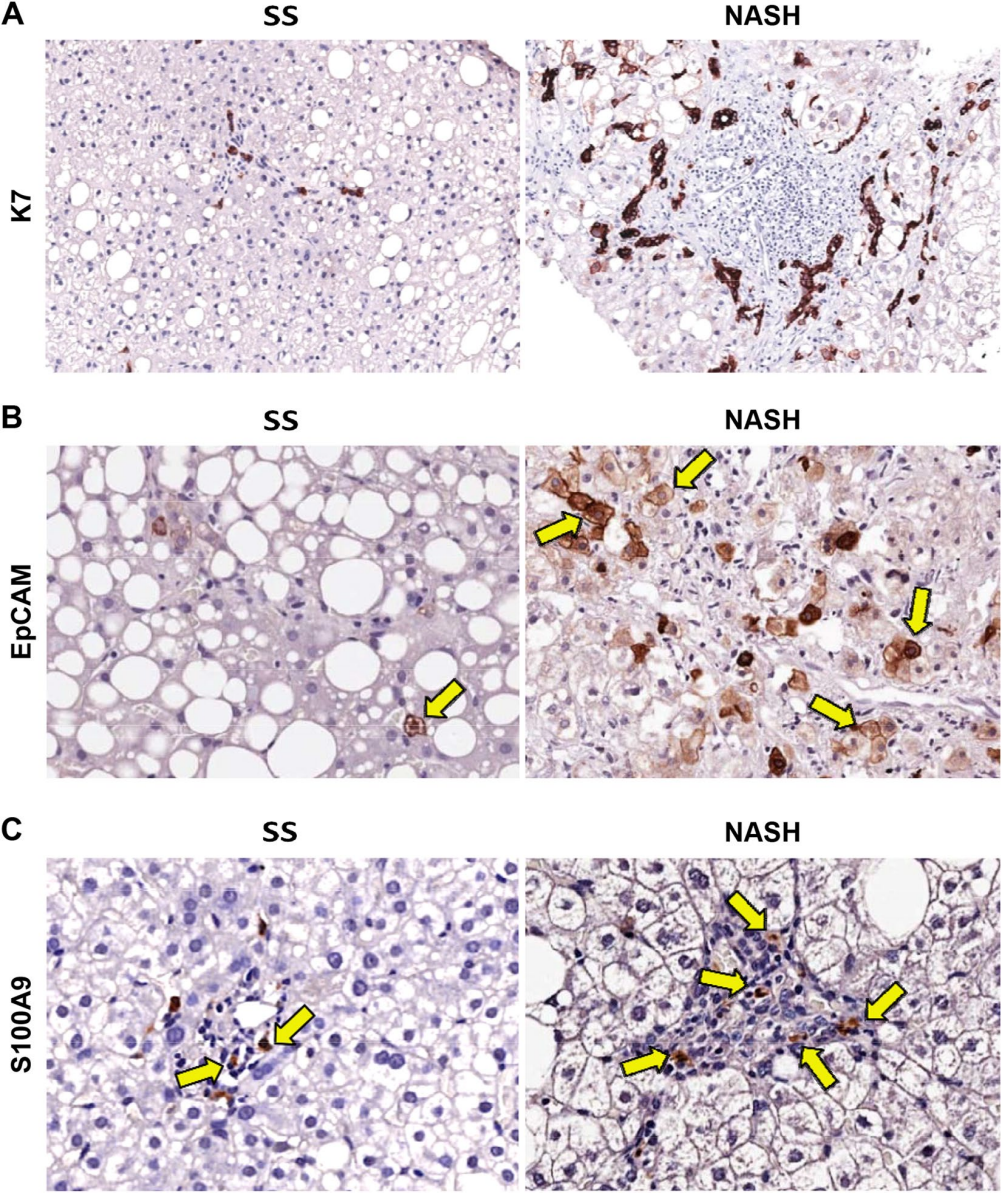
Evaluation of ductular reaction and macrophage pool in non-alcoholic fatty liver disease biopsies. (**A**) Immunohistochemistry for Cytokeratin 7 (K7). Definite steatohepatitis (NASH) biopsies showed more extended ductular reaction if compared with simple steatosis (SS). Original Magnification (OM) = 10x. (**B**) Immunohistochemistry for Epithelial Cell Adhesion Molecule (EpCAM). NASH biopsies showed more numerous EpCAM+ hepatocytes (arrows) if compared with SS. OM = 20x. (**C**) Immunohistochemistry for S100A9. NASH biopsies were characterized by a higher number of S100A9-positive macrophages within the portal spaces (arrows) in comparison with SS. OM = 20x.

When histologic classification was considered (Fig. 2A), DR extension was higher in NASH biopsies when compared with SS ones (Table 2; *p* = 0.005). EpCAM+ hepatocytes were found in 7/25 SS biopsies; contrarily, single occasional or clusters of EpCAM+ hepatocytes were present in 15/25 NASH biopsies (Fig. 2B).

DR extension was significantly correlated with NAS (*r* = 0.469; *p* = 0.001), fibrosis score (*r* = 0.439; *p* = 0.003), and with the presence of EpCAM+ hepatocytes (*r* = 0.578; *p* = 0.000). Furthermore, the presence of EpCAM+ hepatocytes was significantly correlated with NAS score (*r* = 0.509, *p* = 0.000) and fibrosis (*r* = 0.482; *p* = 0.001).

### HSC/MF pool activation

The activation of HSC/MF pool was evaluated by immunohistochemistry for αSMA (Fig. 1). The number of αSMA+ pericentral HSCs was increased in NASH biopsies in comparison with SS ones (Table 2; *p* = 0.024); similarly, the number of αSMA+ portal/septal MFs was increased in NASH in comparison with SS biopsies (Table 2; *p* = 0.034). The number of αSMA+ pericentral HSCs was correlated with the number of αSMA+ portal/septal MFs (*r* = 0.672; *p* = 0.000). Furthermore, the number of αSMA+ pericentral HSCs was correlated with NAS (*r* = 0.353; *p* = 0.014) and fibrosis score (*r* = 0.385; *p* = 0.007) but not with DR. The number of αSMA+ portal/septal MFs was correlated with NAS (*r* = 0.344; *p* = 0.017), fibrosis score (*r* = 0.383; *p* = 0.007), portal inflammation (*r* = 0.357; *p* = 0.013), and DR (*r* = 0.526; *p* = 0.001). Multivariate analysis revealed that the number of αSMA+ portal/septal MFs is independently associated with DR (*p* = 0.028) irrespectively to the other histo-morphological parameters (NAS, fibrosis score, steatosis, lobular inflammation, ballooning, and portal inflammation).

### Macrophage pool

The number of portal but not lobular S100A9+ (pro-inflammatory) macrophages was increased in NASH biopsies in comparison with SS ones (Table 2, *p* = 0.002), when histological diagnosis was taken into consideration (Table 2, Fig. 2C). The number of portal, but not lobular, S100A9+ macrophages was significantly correlated with NAS (*r* = 0.551; *p* = 0.000), fibrosis score (*r* = 0.472; *p* = 0.002), portal inflammation (*r* = 0.511; *p* = 0.001), DR (*r* = 0.479; *p* = 0.004), and the number of αSMA+ portal/septal MFs (*r* = 0.528; *p* = 0.001).

### Histo-pathological and immunohistochemical parameters in PNPLA3 I148M variant carriers

Liver biopsies were classified into wild type homozygous (WT: CC carriers; N = 15) and PNPLA3 I148M variant carriers (PNPLA3 variant carriers: GC/GG carriers; N = 39).

**Figure 3:**
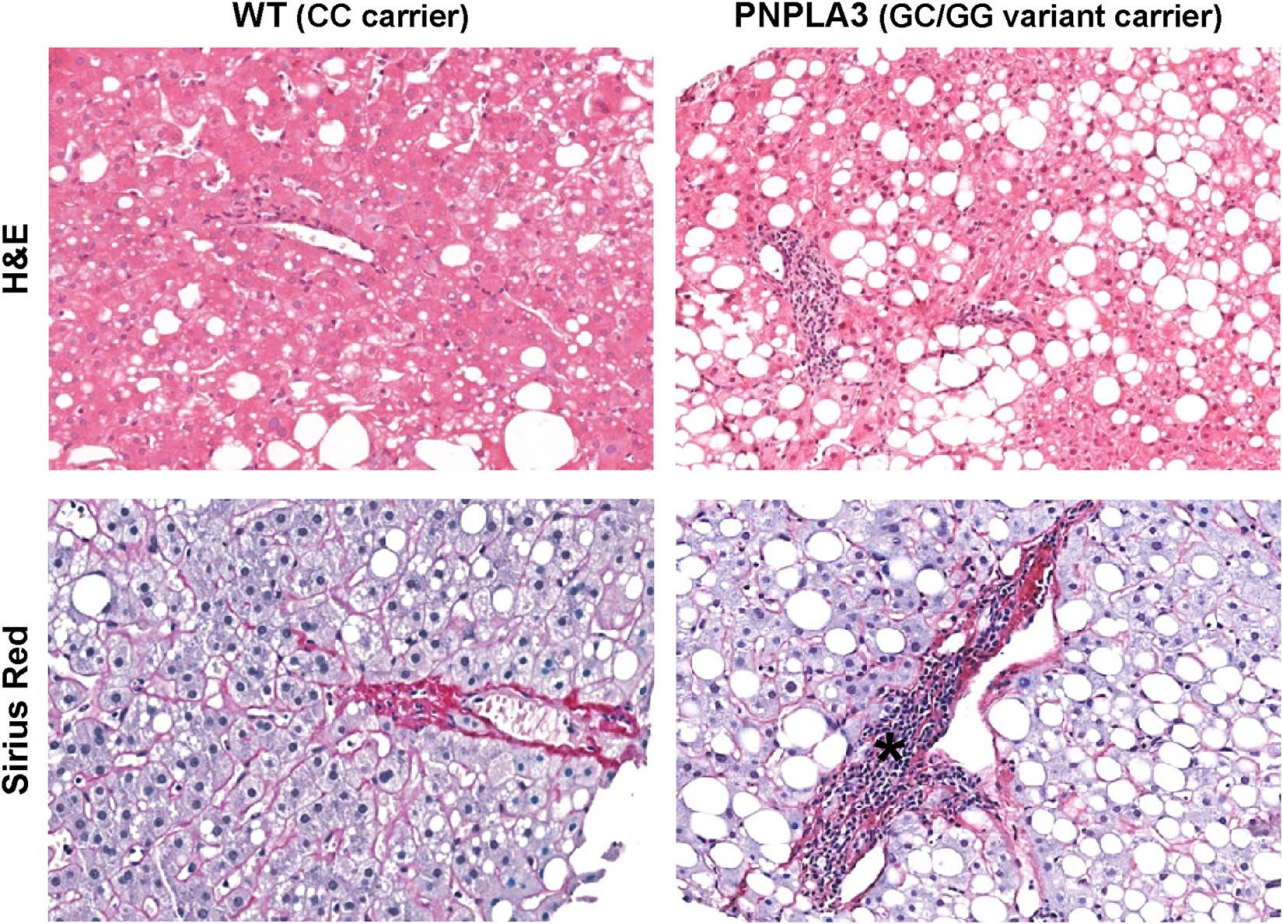
Comparison of histo-morphological aspects between biopsies obtained from patients carrying or non-carrying non-synonymous variant I148M in human patatin-like phospholipase domain containing 3 gene (PNPLA3). Hematoxylin & Eosin (H&E) stain in upper images and Sirius Red stain in lower images. Patients with PNPLA3 variant were characterized by a higher steatosis and portal inflammation (asterisk) in comparison with wild-type (WT) patients. Original Magnification (OM) = 10x.

**Figure 4:**
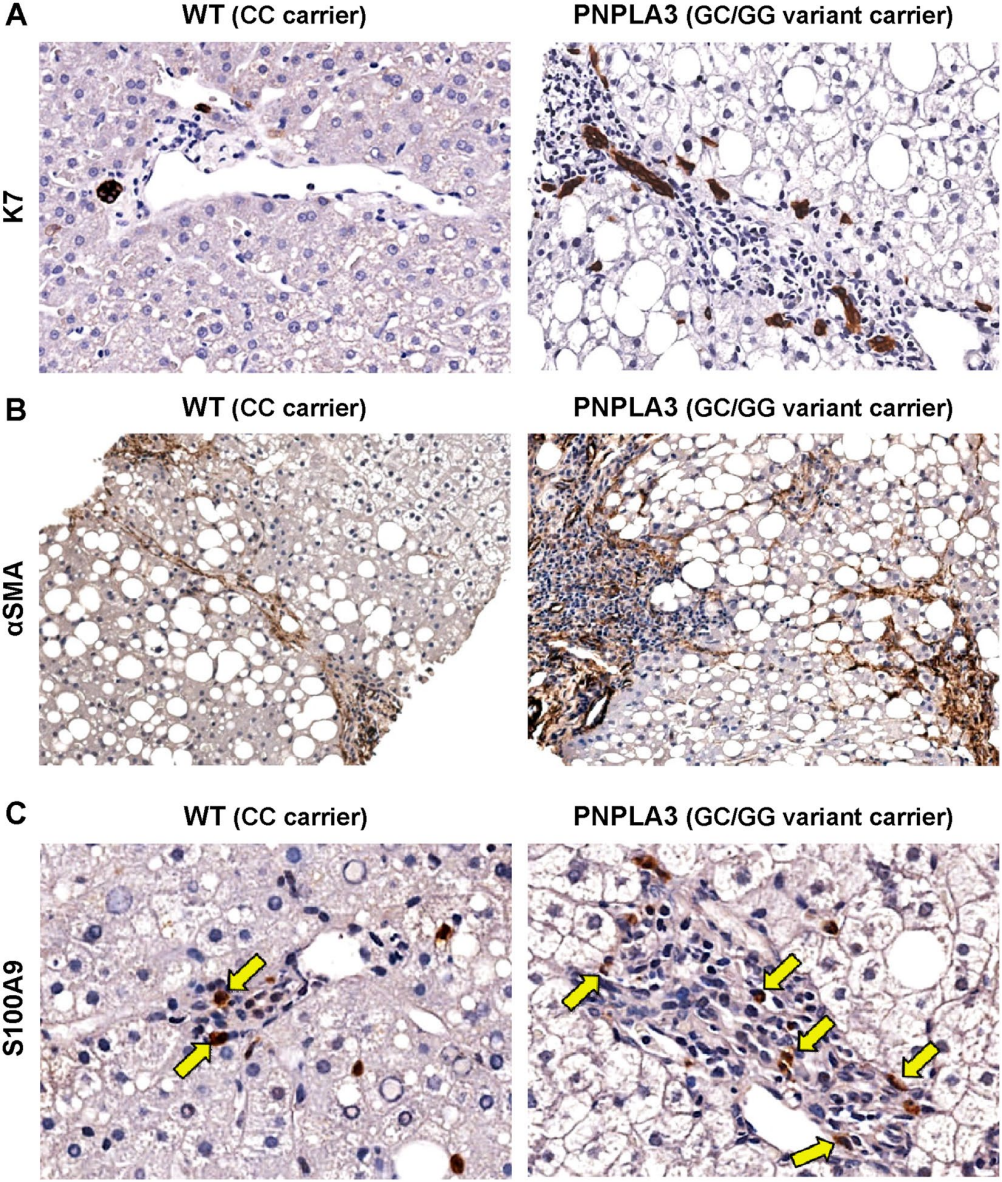
Differences in ductular reaction, hepatic stellate cells/myofibroblasts and macrophage pool between biopsies obtained from patients carrying or non carrying non-synonymous variant I148M in human patatin-like phospholipase domain containing 3 gene (PNPLA3). (**A**) Immunohistochemistry for Cytokeratin 7 (K7). Patients with PNPLA3 variant were characterized by a more extended ductular reaction if compared with WT patients. Original Magnification (OM) = 20x. (**B**) Immunohistochemistry for α smooth muscle actin (αSMA). Higher number of portal/periportal αSMA-positive myofibroblasts was revealed in patients with PNPLA3 variant when compared with WT patients. OM = 10x. (**C**) Immunohistochemistry for S100A9. Patients with PNPLA3 variant were characterized by a higher number of S100A9-positive macrophages within the portal spaces (arrows) in comparison with WT patients. OM = 20x.

No difference between the two groups was present in term of Metabolic Syndrome (MetS) prevalence (Table 3). Overall, there were no differences in disease activity (NAS score) and stage (fibrosis score) when biopsies obtained from WT patients were compared to ones from PNPLA3 variant carriers (Table 3). However, biopsies from PNPLA3 variant carriers showed higher degree of steatosis (*p* = 0.022, Fig. 3), DR (*p* = 0.044, Fig. 4A), IH (*p* = 0.027), and a higher number of αSMA+ portal/septal MFs (*p* = 0.040, Fig. 4B) and portal S100A9+ macrophages (*p* = 0.039, Fig. 4C) when compared with WT patients (Table 3). In biopsies with steatosis >0 obtained from PNPLA3 variant carriers, the steatosis distribution pattern was panacinar in 14, azonal in 10, zone 3 in 8, and zone 1 in 4 cases. Differently, in biopsies with steatosis >0 obtained from WT patients, steatosis distribution pattern was azonal in 5 and zone 3 in 5 cases, and panacinar in 1 case; only 1 biopsy was present in which steatosis was categorized as panacinar or zone 1 (*p* = 0.032 versus PNPLA variant carriers).

**Table 3:** Histological and immunohistochemical findings in NAFLD biopsies obtained from wild type homozygous patients and patients carrying PNPLA3 I148M variant.

	Wild type (CC carrier) N = 15	PNPLA3 (GC/GG variant carrier) N = 39	*p-value*
NAFLD activity score	3.53 ± 1.68	4.15 ± 1.60	0.106
Steatosis	1.13 ± 0.99	1.74 ± 0.97	**0**.**022**
Lobular inflammation	1.33 ± 0.72	1.15 ± 0.94	0.223
Ballooning	1.07 ± 0.80	1.26 ± 0.82	0.401
Portal inflammation (%)	20	59	**0**.**015**
Fibrosis score	1.80 ± 1.08	2.10 ± 0.94	0.157
Ductular Reaction	0.12 ± 0.11	0.38 ± 0.49	**0**.**044**
EpCAM+ hepatocytes	0.33 ± 0.49	0.80 ± 0.76	**0**.**027**
Pericentral αSMA+ HSCs	3.93 ± 3.64	5.64 ± 4.60	0.124
Portal/septal αSMA+ MFs	3.57 ± 3.70	6.47 ± 5.13	**0**.**040**
Lobular S100A9+ macrophages	7.17 ± 3.03	8.14 ± 5.08	0.267
Portal S100A9+ macrophages	2.67 ± 1.91	4.71 ± 3.10	**0**.**039**

Data are reported as mean ± standard deviation. *p value* in bold are statistically significant. PNPLA3: patatin-like phospholipase domain containing 3; HSCs: hepatic stellate cells; MFs: myofibroblasts; αSMA: α smooth muscle actin.

Portal inflammation was seen in 3/15 (20%) WT patients and in 23/39 (59%) PNPLA3 variant carriers (*p* = 0.015). Interestingly, when patients with MetS were separately considered (N = 32), biopsies from PNPLA3 variant carriers (N = 23) still showed significantly higher degree of steatosis (p < 0.05), DR (p < 0.05), and portal inflammation (p < 0.05) when compared with WT patients (N = 9).

Then, liver biopsies were divided according to histologic classification into SS (N = 25) and NASH (N = 25). When SS biopsies were separately considered, PNPLA3 variant carriers showed higher DR extension (*p* = 0.014) and higher number of portal S100A9+ macrophages when compared with WT patients (*p* = 0.020) (Supplementary Table 1). Moreover, in NASH biopsies, PNPLA3 variant carriers showed higher degree of steatosis (*p* = 0.025), portal inflammation (*p* = 0.034), DR (*p* = 0.036), EpCAM+ hepatocytes (*p* = 0.029), and higher number of αSMA+ portal/septal MFs (*p* = 0.011) when compared with WT patients (Supplementary Table 2).

Finally, biopsies from patients carrying PNPLA3 variant (N = 39) were subdivided in two groups according to the presence (N = 23) or absence (N = 16) of MetS. Biopsies from patients with MetS showed higher NAS (*p* = 0.000), lobular inflammation (*p* = 0.007), hepatocellular ballooning (*p* = 0.002), fibrosis score (*p* = 0.001), DR (*p* = 0.007), EpCAM+ hepatocytes (*p* = 0.011), αSMA+ portal/septal MFs (*p* = 0.045), and portal S100A9+ macrophages (*p* = 0.020) when compared with patients without MetS (Table 4).

**Table 4:** Histological and immunohistochemical findings in biopsies from NAFLD patients carrying PNPLA3 I148M variant with or without metabolic syndrome.

	No MetS (n = 16)	MetS (n = 23)	*p-value*
NAFLD activity score	3.19 ± 1.42	4.83 ± 1.37	**0**.**000**
Steatosis	1.56 ± 1.15	1.87 ± 0.81	0.168
Lobular inflammation	0.81 ± 0.75	1.39 ± 0.65	**0**.**007**
Ballooning	0.81 ± 0.75	1.57 ± 0.73	**0**.**002**
Portal inflammation (%)	43.7	69.6	0.100
Fibrosis score	1.56 ± 0.73	2.48 ± 0.90	**0**.**001**
Ductular Reaction	0.15 ± 0.09	0.56 ± 0.59	**0**.**007**
EpCAM+ hepatocytes	0.47 ± 0.51	1.05 ± 0.83	**0**.**011**
Pericentral αSMA+ HSCs	4.35 ± 4.14	6.55 ± 4.78	0.080
Portal/septal αSMA+ MFs	4.77 ± 3.24	7.74 ± 5.94	**0**.**045**
Lobular S100A9+ macrophages	8.95 ± 5.18	7.54 ± 5.05	0.212
Portal S100A9+ macrophages	2.94 ± 1.93	5.56 ± 4.01	**0**.**020**

Data are reported as mean ± standard deviation. *p value* in bold are statistically significant. MetS: metabolic syndrome; HSCs: hepatic stellate cells; MFs, myofibroblasts; αSMA: α smooth muscle actin.

### Serum oxidative stress

To investigate a potential mechanism responsible for the significant difference in histological features observed in NAFLD patients with and without PNPLA3 variant, we measured the activity of Nox2, a key enzyme producing reactive oxidant species that is directly implicated in liver fibrogenesis, and serum F2-Isoprostanes, a validated marker of oxidative stress^15–18^.

Serum Nox2 was correlated with NAS (*r* = 0.59; *p* < 0.001), DR (r = 0.36; *p* < 0.05), the number of αSMA+ pericentral HSCs (*r* = 0.40; *p* < 0.05) and αSMA+ portal/septal MFs (*r* = 0.51; *p* = 0.001), the degree of steatosis (*r* = 0.37; *p* < 0.05) and lobular inflammation (*r* = 0.38; *p* < 0.05). Moreover, serum Nox2 was significantly correlated with hepatocellular ballooning (*r* = 0.36, *p* < 0.05) and portal, but not lobular, S100A9+ macrophages (*r* = 0.42, *p* < 0.05).

Serum F2-isoprostanes were correlated with DR (*r* = 0.38, *p* < 0.05) and portal inflammation (*r* = 0.35; *p* < 0.05). A trend was also found for the number of αSMA+ pericentral HSC (*r* = 0.31; *p* = 0.051) and EpCAM+ hepatocytes (*r* = 0.30, *p* = 0.067).

Serum F2-isoprostanes were significantly higher in I148M variant carriers compared to non-carriers (*p* < 0.05, Table 1).

In a multivariable linear regression model, the association between F2-isoprostanes and I148M variant remained significant (*beta* = 0.34, *p* < 0.05) after adjustment for age, sex, smoking, number of components of MetS, and NAS.

## Discussion

The main findings of the study indicate that NAFLD patients carrying PNPLA3 variant, compared to WT subjects, showed: i) a more prominent portal/periportal pattern in liver damage, ii) a higher activation of HpSC niche, and iii) increased serum oxidative stress levels.

In the present study, patients with and without I148M variant did not differ in term of clinical parameters and histo-pathological staging (fibrosis) and grading (NAS) at liver biopsy. This aspect was essential to allow the comparison of the histologic pattern, HpSC niche activation, and oxidative stress levels, based on the presence/absence of PNPLA3 I148M variant. Patients carrying I148M variant disclosed higher values in hepatocyte steatosis and were characterized by the loss of a predominant pericentral damage location with higher steatosis in periportal hepatocytes and higher portal inflammation compared to WT subjects. The loss of function of lipase activity of PNPLA3 I148M variant promotes triglyceride accumulation, which can initially affect pericentral hepatocytes^2^; this could induce further dynamic adaptation in fatty acid metabolism, resulting in the early progression of steatosis toward zone 1 within liver lobule^19^.

The higher accumulation or subcellular localization of several lipid compounds in PNPLA3 variant carriers may determine increased peroxidation and oxidative stress (lipotoxicity); lipotoxicity determines the increase of hepatocyte cell cycle arrest and apoptosis, thus leading to the subsequent activation of resident progenitor cell niche in NAFLD^8,9^. HpSCs are facultative stem/progenitor cells which become activated in several human liver diseases^14^. In NAFLD, HpSC activation follows the impairment of hepatocyte proliferation capabilities and is supported by a complex niche composed by macrophages, HSCs, and a well-defined extracellular matrix^20^. Interestingly, patients carrying I148M variant showed increased activation of HpSCs compared to WT subjects in terms of higher DR extension and presence of IH^14^. These differences were maintained when patients were divided according histological diagnosis (SS versus NASH), thus confirming their association with genetic variant independently from the diagnosis of NASH. Proliferating HpSCs are able to activate companion cells within the niche (i.e. HSCs and macrophages) via the production of a variety of growth factors, peptides and cytokines^21,22^. This interactions between HpSCs and companion cells establish pro-fibrogenic loops, resulting in progressive fibrosis^14,20,23^. Our results seem to be in accordance with this scenario; in patients carrying I148M variant HpSC activation and macrophage infiltration were already increased in SS and were followed by a more extensive portal myofibroblast activation in NASH. Remarkably, in previous and present studies, HpSC niche activation has been correlated with a worse NAFLD staging and grading, irrespectively to PNPLA3 genotyping^8,9,13,24^. Taken together, our results indicate that the loss of a predominant pericentral pattern of liver damage and the increased HpSC niche activation are key features in patients carrying I148M variant, independently to other clinical and histological parameters. Our finding may explain, at least in part, the more aggressive course of liver disease observed in patients carrying PNPLA3 variant, as patients with portal fibrogenesis are more prone to develop progressive liver disease and liver-related mortality^25–27^.

Interestingly, previous studies indicated that PNPLA3 variants are associated with the severity of liver damage also in non-NAFLD patients including biliary diseases^28^. Thus, in biliary human diseases and in experimental models^29^, fibrosis is associated with biliary proliferation and ductular reaction. These parallel observations seem to suggest a common pathogenetic link between periportal hepatocyte injury and PNPLA3, irrespectively of disease aetiology and based on DR.

In this context, oxidative stress may represent a possible physio-pathologic link between PNPLA3 variant, histological pattern, and HpSC niche activation. Accordingly, our data indicated higher levels of serum oxidative stress markers in patients carrying PNPLA3 I148M variant compared to WT subjects and a significant correlation between HpSC activation and oxidative stress markers (i.e. F2-isoprostanes and Nox2 activity). Oxidative stress can trigger the activation of HpSC niche not only by causing hepatocyte cell cycle arrest but also by modulating the macrophage and HSC activity states^8,9,30,31^. To this latter regard, human quiescent HSCs express low levels of the catalytic subunits Nox2 and Nox1, which are highly up-regulated *in vitro* and *in vivo* from patients with liver fibrosis^32^. Nox mediates the fibrogenic responses to various agonists, including angiotensin II, platelet-derived growth factor leptin, transforming growth factor (TGF)-β, and advanced glycation end products; furthermore, Nox2 is involved in the activation of HSCs following phagocytosis of apoptotic hepatocytes by macrophages^18^. Previous study also showed that F2-isoprostanes generated by lipid peroxidation in hepatocytes mediate HSC proliferation and collagen hyperproduction seen in hepatic fibrosis^33^. Finally, PNPLA3 mutation has been showed to affect HSC activation by acting on vitamin A metabolism and increasing vitamin A retention inside the cells, thus reducing antioxidant availability^34^.

Besides I148M variant and oxidative stress, MetS represents a relevant clinical condition predisposing to NASH development and fibrosis^35^. In keeping, patients with I148M variant and MetS showed a more severe overall damaging, a more advanced staging, and a higher HpSC activation in comparison with patients without MetS.

Finally, it is interesting to note a lower prevalence of arterial hypertension in carriers of the I148M mutation, suggesting that in patients with genetic predisposition, the presence of cardio-metabolic risk factors is not always necessary to develop liver steatosis, as already reported in a large series of individuals with NAFLD^36^.

Our study has clinical and pathophysiologic implications. Patients with PNPLA3 I148M variant represent a subset of NAFLD patients at higher risk for liver damage, characterized by a particularly increased oxidative stress, which seems to play a key role in triggering the process of fibrosis. These patients may particularly benefit from a preventive strategy aimed to reduce oxidative stress and to a tight control of associated cardio-metabolic risk factors.

A limitation of the study is its cross-sectional design, as it does not allow to investigate if oxidative stress is associated with a more rapid/severe development of liver failure over time. An *ad hoc* longitudinal study is needed to clarify these aspects. Moreover, all patients who were included in the study were overweight/obese with a high prevalence of MetS; thus, further studies are needed to extend our findings to lean or normal weight NAFLD patients.

Finally, the association between specific pattern of liver fibrosis and other genetic variants should be further tested, as recent evidence suggested that, TM6SF2 p.E167K, and MBOAT7 rs641738 variants are associated with increased liver steatosis and fibrosis^37^.

In conclusions, NAFLD patients carrying PNPLA3 I148M variant are characterized by a specific histological pattern, higher HpSC niche activation and increased oxidative stress.

## Materials and Methods

### Patients

We included 54 patients with biopsy-proven NAFLD. Anthropometric data (i.e. waist circumference and body mass index, BMI) and information on concomitant treatment and co-morbidities were registered. Routine clinical and biochemical evaluations were obtained for all patients. Inclusion criteria were: no history of current/past excessive alcohol drinking as defined by an average daily consumption of alcohol >20 g; negative tests for the presence of hepatitis B surface antigen and antibody to hepatitis C virus, BMI >24 Kg/m^2^. Exclusion criteria were evidence of chronic, progressive liver disease, active cancer and current supplementation with antioxidants or vitamins. Cardiovascular and metabolic risk factors were defined as follows: arterial hypertension as repeated elevated blood pressure values (≥140/≥90 mmHg) or taking antihypertensive drugs^38^; diabetes as a casual plasma glucose ≥200 mg/dl (11.1 mmol/l), or fasting plasma glucose ≥126 mg/dl (7.0 mmol/l), or presence of anti-diabetic treatment^39^. Metabolic syndrome (MetS) was classified according to the modified criteria of the ATP III Expert Panel of the US-NCEP^40^.

Informed written consent was obtained and the study protocol conformed to the ethical guidelines of the 1975 Declaration of Helsinki as reflected in a priori approval by the local ethical board of Sapienza University of Rome^41^.

### Liver biopsy and histo-pathological analysis

Percutaneous ultrasonography-guided liver biopsy was performed in NAFLD patients with persistent elevation of liver enzymes (>6 months). Liver biopsy was conducted under conscious sedation using a 16-gauge Klatskin needle. Liver fragments were fixed in buffered formalin for 2–4 hours and embedded in paraffin with a melting point of 55 °C–57 °C. Three- to 5-µm sections were cut and stained with hematoxylin and eosin and Sirius Red stains. A minimum biopsy specimen length of 15 mm or at least the presence of five complete portal tracts was required.

Histo-pathological evaluation has been performed on the basis of the NAFLD Clinical Research Network (CRN) criteria^42^. Features of steatosis (0–3), lobular inflammation (0–3), and hepatocyte ballooning (0–2) were combined to obtain the NAFLD activity score (NAS). Fibrosis score (0–4) was assigned based on Sirius Red stains^42^. As recommended^43^, a microscopic diagnosis based on overall injury pattern as well as the presence of additional lesions have been assigned to each case^44^. Biopsies were classified into simple steatosis with not-definite steatohepatitis (SS), definite steatohepatitis (NASH), borderline zone 1 pattern or borderline zone 3 pattern subcategories^43^.

Sections were examined with a Leica Microsystems DM 4500 B Microscopy (Weltzlar, Germany) equipped with a Jenoptik Prog Res C10 Plus Videocam (Jena, Germany) and were processed with an IAS—Delta Sistemi (Milan, Italy) and were independently scored by two researchers in double blind fashion.

### Immunohistochemistry

For immunohistochemistry, endogenous peroxidase activity was blocked by a 30-min incubation in methanolic hydrogen peroxide (2.5%). Antigens were retrieved, as indicated by the vendor, by applying Proteinase K (Dako, Glostrup, Denmark, code S3020) for 10 min at room temperature. Sections were then incubated overnight at 4 °C with primary antibodies against cytokeratin 7 (K7: mouse monoclonal; code: M7018; diluition: 1:50; Dako, Glostrup, Denmark), EpCAM (Dako Glostrup, Denmark, mouse monoclonal, code: M3525, dilution: 1:100), α Smooth Muscle Actin (αSMA: mouse monoclonal; code M0851; diluition 1:50; Dako, Glostrup, Denmark), and S100A9 (rabbit monoclonal; code: ab92507; diluition 1:200; Abcam, Cambridge, United Kingdom).

Then, samples were rinsed twice with phosphate buffered saline (PBS) for 5 minutes, incubated for 20 minutes at room temperature (RT) with secondary biotinylated antibody, and then with Streptavidin-horseradish peroxidase (LSAB+ , Dako, Glostrup, Denmark code K0690). Diaminobenzidine (Dako, Glostrup, Denmark code K3468) was used as substrate, and sections were counterstained with haematoxylin. For all immunoreactions, negative controls (the primary antibody was replaced with pre-immune serum) were also included^45^.

Slides were scanned by a digital scanner (Aperio Scanscope CS System, Aperio Digital Pathology, Leica Biosystems, Milan, Italy) and processed by ImageScope^46^. Only biopsies containing at least five portal spaces were considered.

The degree of HpSC activation was evaluated by the extension of ductular reaction (DR). The area occupied by DR was evaluated by K7 immunoreactivity, quantified by an image analysis algorithm, and was expressed as the percentage of the parenchymal area occupied by reactive ductules, as previously^13^. Cholangiocytes lining the interlobular bile ducts were excluded from the counts.

To assess the commitment of progenitor cells toward a hepatocyte fate, the presence of EpCAM+ hepatocytes has been investigated by immunohistochemistry. EpCAM+ hepatocytes have been shown to represent the progeny of stem/progenitor cells within bile ductules. The presence of EpCAM+ hepatocytes was scored as: 0 = no positive cells, 1 (level 1) = single occasional, and 2 (level 2) = clusters of EpCAM+ hepatocyte^13^.

The activation of Hepatic Stellate Cell (HSC)/Myofibroblast (MF) pool was evaluated by counting the number of αSMA-positive cells per high power field (HPF: at 40x). Perisinusoidal HSCs and portal/septal MFs were separately evaluated; αSMA-positive HSCs were recognized in accordance with their stellate/spindle shape and their perisinusoidal location within the parenchymal lobule; besides, portal/septal MFs were considered as stellate- or spindle-shaped αSMA-positive cells located at the interface between parenchyma and portal tract or between parenchyma and septa, and those residing in the portal tracts and the fibrotic septa. The number of αSMA-positive HSCs and MFs was counted and expressed as number of positive cells per HPF. For each slide, at least 10 non-overlapping microscopic HPFs were randomly chosen^47^.

The presence of macrophages with an inflammatory phenotype was calculated as the number of S100A9+ cells per HPF. For each slide, at least 10 non-overlapping microscopic HPFs were randomly chosen^13^.

### Serum Nox2

To quantify NOX2 activity, we measured serum levels of soluble NOX2-derived peptide (sNOX2-dp), a marker of NOX2 activation, by ELISA method as previously described^48^ Blood samples were kept for 60 minutes at 37 °C and centrifuged at 300 g; serum was stored at −80 °C. Values were expressed as picograms per milliliter; intra-assay and inter-assay coefficients of variation were 5.2% and 6%, respectively.

### Serum 8-isoprostaglandin F_2α_

Serum 8-isoprostaglandin F_2α_ (8-isoPGF_2α_, F2-isoprostanes) levels were assessed by a previously described and validated EIA assay method and expressed as pg/ml^49^. Intra- and inter-assay coefficients of variation were 5.8% and 5.0% respectively.

### Analysis of PNPLA3

DNA was extracted from peripheral blood and purified by the Wizard® Genomic DNA Purification Kit following the manufacturing protocol. Fluorogenic 5′-nucleotidase assays were developed to genotype the *PNPLA3* rs738409 C to G non-synonymous sequence variant, encoding I148M, in all subjects. The assay was performed using the TaqMan C7241_10 assay (Applied Biosystems, Foster City, CA) on ABI PRISM 7900 HT Sequence Detection Systems. (Applied Biosystems, Foster City, CA). The plate was run using standard condition at 95 C for 10 min, 95 C for 15 s then 60 °C for 1 min for 40 cycles. Allele frequencies were in Hardy–Weinberg equilibrium. The TaqMan assay was validated by direct sequencing of the SNP (rs738409) in representative samples of DNA on ABI PRISM 3130 XL Genetic Analyzer, and both methods gave identical results.

### Statistical Analysis

Categorical variables were reported as counts (percentage). Continuous variables were expressed as mean ± standard deviation or median and interquartile range. Normal distribution of parameters was assessed by Kolmogorov–Smirnov test. Student unpaired t test and Pearson correlation analysis were used for normally distributed continuous variables. Appropriate nonparametric tests (Mann-Whitney U test and Spearman rank correlation test) were employed for all the other variables. Categorical variables were compared using the Chi-squared test or Fischer’s Exact test as appropriate. All tests were two-tailed and a statistical significance was set at a *p* value of less than 0.05. Analyses were performed using computer software packages (IBM SPSS Statistics v20.0, Armonk, NY).

### Sample size calculation

We computed the minimum sample size with respect to a two-tailed one-sample Student’s t-test, considering (i) as significant a difference in F2-isoprostanes of 8 pg/ml, (ii) a standard deviation of the paired differences of 6 pg/ml%, (iii) a type I error probability α = 0.05 and power 1-β = 0.90. This resulted in n = 24 (12 for each group).

## Electronic supplementary material

Supplementary data

## References

[1] Francque, S. M., van der Graaff, D. & Kwanten, W. J. Non-alcoholic fatty liver disease and cardiovascular risk: Pathophysiological mechanisms and implications. *J Hepatol*, 10.1016/j.jhep.2016.04.005 (2016). 10.1016/j.jhep.2016.04.005 27091791

[2] Trepo E, Romeo S, Zucman-Rossi J, Nahon P (2016). PNPLA3 gene in liver diseases. J Hepatol.

[3] Xu R, Tao A, Zhang S, Deng Y, Chen G (2015). Association between patatin-like phospholipase domain containing 3 gene (PNPLA3) polymorphisms and nonalcoholic fatty liver disease: a HuGE review and meta-analysis. Scientific reports.

[4] Rotman Y (2010). The association of genetic variability in patatin-like phospholipase domain-containing protein 3 (PNPLA3) with histological severity of nonalcoholic fatty liver disease. Hepatology.

[5] Sookoian S, Pirola CJ (2011). Meta-analysis of the influence of I148M variant of patatin-like phospholipase domain containing 3 gene (PNPLA3) on the susceptibility and histological severity of nonalcoholic fatty liver disease. Hepatology.

[6] Valenti L (2010). Homozygosity for the patatin-like phospholipase-3/adiponutrin I148M polymorphism influences liver fibrosis in patients with nonalcoholic fatty liver disease. Hepatology.

[7] Carpino G, Renzi A, Onori P, Gaudio E (2013). Role of hepatic progenitor cells in nonalcoholic fatty liver disease development: cellular cross-talks and molecular networks. International journal of molecular sciences.

[8] Nobili V (2012). Hepatic progenitor cells activation, fibrosis and adipokines production in pediatric nonalcoholic fatty liver disease. Hepatology.

[9] Richardson MM (2007). Progressive fibrosis in nonalcoholic steatohepatitis: association with altered regeneration and a ductular reaction. Gastroenterology.

[10] Skoien R (2013). Heterogeneity of fibrosis patterns in non-alcoholic fatty liver disease supports the presence of multiple fibrogenic pathways. Liver Int.

[11] Sakaguchi S, Takahashi S, Sasaki T, Kumagai T, Nagata K (2011). Progression of alcoholic and non-alcoholic steatohepatitis: common metabolic aspects of innate immune system and oxidative stress. Drug Metab Pharmacokinet.

[12] Wan J (2013). M2 Kupffer cells promote M1 Kupffer cell apoptosis: A protective mechanism against alcoholic and non-alcoholic fatty liver disease. Hepatology.

[13] Carpino G (2016). Macrophage Activation in Pediatric Nonalcoholic Fatty Liver Disease (NAFLD) Correlates with Hepatic Progenitor Cell Response via Wnt3a Pathway. PLoS One.

[14] Lanzoni G, Cardinale V, Carpino G (2016). The hepatic, biliary, and pancreatic network of stem/progenitor cell niches in humans: A new reference frame for disease and regeneration. Hepatology.

[15] Pignatelli P (2011). Inherited human gp91phox deficiency is associated with impaired isoprostane formation and platelet dysfunction. Arteriosclerosis, thrombosis, and vascular biology.

[16] Polimeni L (2015). Oxidative stress: New insights on the association of non-alcoholic fatty liver disease and atherosclerosis. World journal of hepatology.

[17] Crosas-Molist E, Fabregat I (2015). Role of NADPH oxidases in the redox biology of liver fibrosis. Redox Biol.

[18] Paik YH (2014). Role of NADPH oxidases in liver fibrosis. Antioxidants & redox signaling.

[19] Hijmans BS, Grefhorst A, Oosterveer MH, Groen AK (2014). Zonation of glucose and fatty acid metabolism in the liver: mechanism and metabolic consequences. Biochimie.

[20] Carpino G (2016). Stem/Progenitor Cell Niches Involved in Hepatic and Biliary Regeneration. Stem Cells Int.

[21] Boulter L (2012). Macrophage-derived Wnt opposes Notch signaling to specify hepatic progenitor cell fate in chronic liver disease. Nat Med.

[22] Boulter L, Lu WY, Forbes SJ (2013). Differentiation of progenitors in the liver: a matter of local choice. J Clin Invest.

[23] Williams MJ, Clouston AD, Forbes SJ (2014). Links between hepatic fibrosis, ductular reaction, and progenitor cell expansion. Gastroenterology.

[24] Della Corte C (2016). Docosahexanoic Acid Plus Vitamin D Treatment Improves Features of NAFLD in Children with Serum Vitamin D Deficiency: Results from a Single Centre Trial. PLoS One.

[25] Ekstedt M (2006). Long-term follow-up of patients with NAFLD and elevated liver enzymes. Hepatology.

[26] Younossi ZM (2011). Pathologic criteria for nonalcoholic steatohepatitis: interprotocol agreement and ability to predict liver-related mortality. Hepatology.

[27] Shibayama Y, Nakata K (1990). The relation of periportal fibrosis to portal hypertension. Journal of hepatology.

[28] Krawczyk M, Grunhage F, Zimmer V, Lammert F (2011). Variant adiponutrin (PNPLA3) represents a common fibrosis risk gene: non-invasive elastography-based study in chronic liver disease. Journal of hepatology.

[29] Sato, K., Meng, F., Giang, T., Glaser, S. & Alpini, G. Mechanisms of cholangiocyte responses to injury. *Biochimica et biophysica acta*, 10.1016/j.bbadis.2017.06.017 (2017). 10.1016/j.bbadis.2017.06.017 PMC5742086 28648950

[30] Gouw AS, Clouston AD, Theise ND (2011). Ductular reactions in human liver: diversity at the interface. Hepatology.

[31] Forbes SJ, Newsome PN (2016). Liver regeneration - mechanisms and models to clinical application. Nat Rev Gastroenterol Hepatol.

[32] Bataller R (2003). NADPH oxidase signal transduces angiotensin II in hepatic stellate cells and is critical in hepatic fibrosis. The Journal of clinical investigation.

[33] Comporti M (2008). Isoprostanes and hepatic fibrosis. Molecular aspects of medicine.

[34] Pingitore, P. *et al*. PNPLA3 overexpression results in reduction of proteins predisposing to fibrosis. *Hum Mol Genet*, 10.1093/hmg/ddw341 (2016). 10.1093/hmg/ddw341 PMC5886043 27742777

[35] Anstee QM, Seth D, Day CP (2016). Genetic Factors That Affect Risk of Alcoholic and Nonalcoholic Fatty Liver Disease. Gastroenterology.

[36] Del Ben M (2014). Non-alcoholic fatty liver disease, metabolic syndrome and patatin-like phospholipase domain-containing protein3 gene variants. European journal of internal medicine.

[37] Krawczyk M (2017). Combined effects of the PNPLA3rs738409, TM6SF2 rs58542926, and MBOAT7 rs641738 variants on NAFLD severity: a multicenter biopsy-based study. Journal of lipid research.

[38] Mancia G (2013). 2013 Practice guidelines for the management of arterial hypertension of the European Society of Hypertension (ESH) and the European Society of Cardiology (ESC): ESH/ESC Task Force for the Management of Arterial Hypertension. Journal of hypertension.

[39] Authors/Task Force M (2013). ESC Guidelines on diabetes, pre-diabetes, and cardiovascular diseases developed in collaboration with the EASD: The Task Force on diabetes, pre-diabetes, and cardiovascular diseases of the European Society of Cardiology (ESC) and developed in collaboration with the European Association for the Study of Diabetes (EASD). European heart journal.

[40] Grundy SM (2005). Diagnosis and management of the metabolic syndrome: an American Heart Association/National Heart, Lung, and Blood Institute Scientific Statement. Circulation.

[41] World Medical, A. World Medical Association Declaration of Helsinki. Ethical principles for medical research involving human subjects. *Bulletin of the World Health Organization* **79**, 373–374 (2001). PMC2566407 11357217

[42] Kleiner DE (2005). Design and validation of a histological scoring system for nonalcoholic fatty liver disease. Hepatology.

[43] Brunt EM (2011). Nonalcoholic fatty liver disease (NAFLD) activity score and the histopathologic diagnosis in NAFLD: distinct clinicopathologic meanings. Hepatology.

[44] Brunt EM (2001). Nonalcoholic steatohepatitis: definition and pathology. Semin Liver Dis.

[45] Onori P (2007). Activation of the IGF1 system characterizes cholangiocyte survival during progression of primary biliary cirrhosis. The journal of histochemistry and cytochemistry: official journal of the Histochemistry Society.

[46] Carpino G (2014). Evidence for multipotent endodermal stem/progenitor cell populations in human gallbladder. J Hepatol.

[47] Carpino G (2005). Alpha-SMA expression in hepatic stellate cells and quantitative analysis of hepatic fibrosis in cirrhosis and in recurrent chronic hepatitis after liver transplantation. Dig Liver Dis.

[48] Pignatelli P (2010). Atorvastatin inhibits gp91phox circulating levels in patients with hypercholesterolemia. Arteriosclerosis, thrombosis, and vascular biology.

[49] Hoffman SW, Roof RL, Stein DG (1996). A reliable and sensitive enzyme immunoassay method for measuring 8-isoprostaglandin F2 alpha: a marker for lipid peroxidation after experimental brain injury. J Neurosci Methods.

