# Supplementary material for: PNPLA3 variant and portal/periportal histological pattern in patients with biopsy-proven non-alcoholic fatty liver disease: a possible role for oxidative stress
Source: Sci Rep. 2017 Nov 17;7:15756. doi: 10.1038/s41598-017-15943-z (PMC5693899; doi:10.1038/s41598-017-15943-z)
Supplement: Supplementary file 1 — Supplementary data [file 41598_2017_15943_MOESM1_ESM.doc]

**Supplementary data file**

**PNPLA3 variant and portal/periportal histological pattern in patients with biopsy-proven non-alcoholic fatty liver disease: a possible role for oxidative stress**

Guido Carpino^1^*, Daniele Pastori^2,3^*, Francesco Baratta^2,3^, Diletta Overi^3^, Giancarlo Labbadia^2^, Licia Polimeni^2^, Alessia Di Costanzo^2^, Gaetano Pannitteri^4^, Roberto Carnevale^2,5^, Maria Del Ben^2^, Marcello Arca^2^, Francesco Violi^2^, Francesco Angelico^6^ and Eugenio Gaudio^3^.

*equal contribution

**Affiliations:**

^1^Department of Movement, Human and Health Sciences, University of Rome "Foro Italico", Rome, Italy. ^2^Department of Internal Medicine and Medical Specialties, I Clinica Medica, Sapienza University of Rome, Italy. ^3^Department of Anatomical, Histological, Forensic Medicine and Orthopaedics Sciences, Sapienza University, Rome, Italy. ^4^Department of Cardiovascular, Respiratory, Nephrologic, Anaesthesiologic and Geriatric Sciences, Sapienza University of Rome, Rome, Italy.^5^Department of Medical-Surgical Sciences and Biotechnologies, Sapienza University of Rome, Latina, Italy. ^6^Department of Public Health and Infectious Diseases, Sapienza University, Rome, Italy.

**Corresponding author:** Prof. Francesco Angelico. Department of Public Health and Infectious Disease, Sapienza University, Rome, Italy. I Clinica Medica – Policlinico Umberto I. Viale del Policlinico 155, 00161 Rome, Italy. Phone/fax +39 9649972249. Email: [francesco.angelico@uniroma1.it](mailto:francesco.angelico@uniroma1.it)

**Supplementary Table 1. Histological and immunohistochemical findings in Simple Steatosis biopsies obtained from wild type homozygous (WT) patients and patients carrying PNPLA3 I148M variant.**

|  | **WT**  (CC carrier)  N= 8 | **PNPLA3**  (GC/GG variant carrier)  N=17 | ***p-value*** |
| --- | --- | --- | --- |
| **NAS** | 2.50±1.07 | 2.82±1.01 | 0.236 |
| **Steatosis** | 0.75±0.89 | 1.18±0.88 | 0.136 |
| **Lobular inflammation** | 0.87±0.64 | 0.65±0.49 | 0.168 |
| **Ballooning** | 0.88±0.83 | 1.00±0.79 | 0.360 |
| **Portal inflammation (%)** | 12.5 | 41.1 | 0.205 |
| **Fibrosis score** | 1.12±0.99 | 1.37±0.68 | 0.233 |
| **Ductular Reaction** | 0.06±0.06 | 0.19±0.13 | **0.014** |
| **EpCAM+ hepatocytes** | 0.17±0.41 | 0.50±0.63 | 0.123 |
| **Pericentral HSCs** | 2.01±0.77 | 4.21±4.20 | 0.133 |
| **Portal/septal MFs** | 4.61±4.60 | 4.55±3.35 | 0.486 |
| **Lobular S100A9+ macrophages** | 6.86±3.36 | 9.29±5.90 | 0.197 |
| **Portal S100A9+ macrophages** | 0.93±0.87 | 2.81±1.47 | **0.020** |
| **Metabolic Syndrome (%)** | 37.5 | 29.4 | 0.513 |

Data are reported as mean ± standard deviation. *p value* in bold are statistically significant. SS, simple steatosis; NAS, NAFLD activity score; HSCs, hepatic stellate cells; MFs, myofibroblasts.

**Supplementary Table 2. Histological and immunohistochemical findings in NASH biopsies obtained from wild type homozygous (WT) patients and patients carrying PNPLA3 I148M variant.**

|  | **WT**  (CC carrier)  N= 7 | **PNPLA3**  (GC/GG variant carrier)  N=18 | ***p-value*** |
| --- | --- | --- | --- |
| **NAS** | 4.71±1.50 | 5.44±1.04 | 0.089 |
| **Steatosis** | 1.57±0.98 | 2.33±0.77 | **0.025** |
| **Lobular inflammation** | 1.86±0.38 | 1.61±0.70 | 0.195 |
| **Ballooning** | 1.29±0.75 | 1.50±0.79 | 0.271 |
| **Portal inflammation (%)** | 28.5 | 77.7 | **0.034** |
| **Fibrosis score** | 2.57±0.53 | 2.72±0.83 | 0.330 |
| **Ductular Reaction** | 0.19±0.12 | 0.83±0.71 | **0.036** |
| **EpCAM+ hepatocytes** | 0.5±0.55 | 1.2±0.77 | **0.029** |
| **Pericentral HSCs** | 4.01±2.93 | 6.35±4.79 | 0.148 |
| **Portal/septal MFs** | 2.54±2.53 | 8.87±5.76 | **0.011** |
| **Lobular S100A9+ macrophages** | 7.39±3.02 | 7.32±4.30 | 0.486 |
| **Portal S100A9+ macrophages** | 4.05±1.16 | 6.24±3.65 | 0.11 |
| **Metabolic Syndrome (%)** | 86 | 83 | 0.693 |

Data are reported as mean ± standard deviation. *p value* in bold are statistically significant. NASH, nonalcoholic steatohepatitis; NAS, NAFLD activity score; HSCs, hepatic stellate cells; MFs, myofibroblasts.
